# Prevalence and associated comorbidities of restless legs syndrome (RLS): Data from a large population-based door-to-door survey on 19176 adults in Tehran, Iran

**DOI:** 10.1371/journal.pone.0172593

**Published:** 2017-02-17

**Authors:** Seyed-Mohammad Fereshtehnejad, Arash Rahmani, Mahdiyeh Shafieesabet, Mahshid Soori, Ahmad Delbari, Mohammad Reza Motamed, Johan Lökk

**Affiliations:** 1 Division of Clinical geriatrics, Department of Neurobiology, Care Sciences, and Society (NVS), Karolinska Institutet, Stockholm, Sweden; 2 Department of Neurology and Neurosurgery, Faculty of Medicine, McGill University, Montreal General Hospital, Montreal, Québec, Canada; 3 Firoozgar Clinical Research Development Center (FCRDC), Firoozgar Hospital, Iran University of Medical Sciences, Tehran, Iran; 4 Medical Student Research Committee (MSRC), Mental Health Research Center, Tehran Psychiatry Institute, Iran University of Medical Sciences, Tehran, Iran; 5 Medical Student Research Committee (MSRC), Faculty of Medicine, Iran University of Medical Sciences, Tehran, Iran; 6 Iranian Research Center on Aging, University of Social Welfare and Rehabilitation, Tehran, Iran; 7 Neurology Department, Faculty of Medicine, Iran University of Medical Sciences, Tehran, Iran; 8 Department of Geriatric Medicine, Karolinska University Hospital, Stockholm, Sweden; University of Florida, UNITED STATES

## Abstract

**Background:**

Discrepancies have been reported in the prevalence rate of restless legs syndrome (RLS) among different ethnic groups and geographic populations. Furthermore, there are disagreements on determinant factors and associated comorbidities of RLS. We aimed to estimate prevalence of RLS and investigate its associated comorbid conditions and risk factors in a large population-based door-to-door survey.

**Methods:**

Following a multistage random sampling from the households lived in 22 urban districts of Tehran, Iran, 19176 participants with ≥30 years of age were recruited. Trained surveyors filled study checklist consisting of baseline characteristics, risk factors and comorbidity profile and the International RLS Study Group (IRLSSG) diagnostic criteria through face-to-face interviews.

**Results:**

In total, 1580 individuals were positively screened for RLS resulting in a standardized prevalence rate of 60.0/1000. There was a gradual increase in RLS prevalence by advancing age, however, sex difference disappeared after adjustment. Parkinsonism [adjusted odds’ ratio (adj-OR) = 7.4 (95% CI: 5.3–10.4)], peripheral neuropathy [adj-OR = 3.7 (95% CI: 3.3–4.1)], subjective cognitive impairment (SCI) [adj-OR = 3.1 (95% CI: 2.7–3.4)], acting out dreams [adj-OR = 2.8 (95% CI: 2.5–3.2)], hyposmia [adj-OR = 2.5 (95% CI: 2.2–2.9)], active smoking [adj-OR = 1.5 (95% CI: 1.3–1.9)] and additional number of cardiometabolic diseases associated with higher risk of RLS [adj-OR = 1.6 (95% CI: 1.2–2.3)].

**Conclusion:**

Our findings showed that neuro-cognitive co-morbidities such as parkinsonism, peripheral neuropathy, SCI, acting out dreams and hyposmia as well as cardio-metabolic risk factors and diseases were independent determinants of RLS. It is recommended to screen individuals with either these comorbid conditions for RLS or the ones with RLS for the accompanying diseases.

## Introduction

Restless legs syndrome (RLS), also known as Willis–Ekbom disease, is a common sensorimotor neurological syndrome which is clinically characterized by unpleasant sensations such as itching, creeping and tingling in limbs, particularly in legs. The symptoms occur mostly at rest and provoke an irresistible urge to move legs [1]. In most epidemiological studies, RLS is diagnosed according to the minimal diagnostic criteria of the international restless legs syndrome study group (IRLSSG). Prevalence of RLS has been estimated to vary between 3.9% and 14.3% in general populations and generally increases by age [2]. Previous investigations have shown that RLS is linked to other mental and somatic disorders such as depression, anxiety [3], cardiovascular disorders [4, 5], chronic pain [6] and decreased cognitive functioning [7].

Although RLS has devastating effects on quality of life [8], early correct diagnosis and providing appropriate interventions to improve RLS are still vastly ignored from both the patients and healthcare professionals [9]. This under-diagnosis might stem from our lack of knowledge about the epidemiologic characteristics of RLS in each society. Epidemiological studies have reported discrepancies in the prevalence of RLS among different ethnic groups and geographic populations. These surveys have reported dissimilar findings regarding the role of sex and age in the incidence of RLS, and its comorbidity profile in different Western and Asian populations [10]. Such a conspicuous variety is probably in part due to methodological issues such as definition of RLS, sampling frame and method for recruiting participants, and list of environmental factors and ethno-demographic determinants.

Dearth of knowledge about the prevalence of RLS, its associated co-morbidities and risk factors in Iranian population and the greater Eastern Mediterranean region, all necessitate the performance of an appropriate epidemiologic survey. We aimed to ascertain prevalence of RLS and identify some potential risk factors and co-morbidities related to RLS in Iranian population using a community-based door-to-door survey.

## Materials and methods

### Study setting

This Population-based door-to-door survey was performed in the metropolitan area of Tehran, Iran consisting of 22 urban districts during October 2011 and January 2012. Present study is a part of a larger project designed to study the epidemiologic features of the major neurological disorders, which has been previously described [11].

### Ethical considerations

The research ethics committee of the *Firoozgar Clinical Research Development Center (FCRDC)* (affiliated to Iran University of Medical Sciences) has approved the study protocol. All participants were informed about the objectives when they were contacted at doors and they provided their verbal consent prior to recruitment. Since the study was designed as an observational research without any extra intervention, verbal consent was satisfactory for the above-mentioned regional research committee. Participation was voluntary and surveyors were trained and informed not to fill the initial checklists if any individual refused to take part and/or didn’t provide the consent.

### Sampling method

In order to have a representative study population, we applied a probability multistage sampling method considering each of the 22 urban districts of Tehran as one stratum with hierarchical socioeconomic status. Afterwards, residential blocks and households were labeled as sampling clusters within each district and were randomly selected by the network of 374 “*Health Centers*” organized by the health deputy of Tehran municipality.

### Data collection and study population

Prior to data collection, a one-day workshop was held to train all surveyors who were among the healthcare workers of each district employed by the “Health Centers”. The workshop aimed to ensure that the surveyors know how to interview and communicate with study participants, to assess their capability to understand items’ definitions and valid answers, to comprehend study instructions, sampling method, managing non-response cases, daily report and field supervision, and other details required for conducting the survey. Following random selection of each household by the surveyors, all family members were asked to participate if they aged ≥30 year. Data collection was done through face-to-face interviews to fill the self-reported checklist. Overall, 20621 individuals answered both baseline checklist and screening questionnaire. After data preparation and exclusion of the ones with missing information on the key variables, data from 19176 participants were entered in the final analysis.

### Variables and instruments

As previously described [11, 12], we used a checklist consisted of three main sections: baseline characteristics [age, sex, level of education, marriage, working status and menopause (in women)], comorbidity profile, and screening questions for the symptoms of selected neurologic disorders. For baseline demographics, smoking was considered positive if the interviewee had either previous history of smoking or was a current smoker. Level of education was categorized into five ordinal levels as: illiterate, primary school, secondary school, high school/diploma, college/university education. Marriage status was defined as either single, married, widow, or divorced. Working status was recorded as either of these five conditions: unemployed, employed, paid without working, housekeeper, retired or others.

For the screening section, we merged items from several different questionnaires: the Sicilian neuro-epidemiology study (SNES) [13], the Baylor Health Screening Questionnaire (BHSQ) [14], telephone questionnaire for Parkinson’s disease [15], the questionnaires either developed or modified by Tanner [16], Daurate [17], Chan [18], Setthawatcharawanich [19] and Sevillano [20] and the WHO screening instrument to measure the prevalence of neurological disabilities in resource-poor settings without registry-based data [21]. Of note, from the list of repeated questions/items from different original questionnaires on the same symptom/entity, only one single question was included in the merged checklist. Other than RLS as the main disease of interest, neurologic disorders that were investigated in our study were stroke, headache, back pain, seizure, parkinsonism, acting out dreams (as one sleep problem), subjective cognitive impairment (SCI), peripheral neuropathy and hyposmia. Presence of these entities/diseases was determined by the standard questions/items that were embedded in the above-mentioned screening questionnaires. In order to screen parkinsonism, we used our previously validated list of questions [12]. We also defined a new variable, “cardiometabolic burden”, by summing up the number of cardiometabolic diseases consisting of heart failure, hypertension, hyperlipidemia, diabetes and stroke ranging from 0 to 5.

The complete case-wise data sheet is provided as supplementary material (S1 Data).

### Definition of RLS

We used the International RLS Study Group (IRLSSG) consensus diagnostic features [22] for screening of RLS. Individuals who met all the following four criteria were considered as RLS+:

1. An urge to move limbs, which usually accompanied or caused by uncomfortable and unpleasant feelings in the limbs
2. Worsening of symptoms with rest or inactivity
3. Improvement of the urge to move by getting up or moving
4. Worsening at the evenings or nighttime appearance of the symptoms [22]

Of note, we asked for the lifetime occurrence of the symptoms during the interview.

### Statistical analysis

Numeric and categorical variables were described as mean [standard deviation (SD)] and frequency (percentage), respectively. We adjusted the prevalence rate of RLS based on the real age and sex distribution of Tehran population according to the national census. Univariate comparisons between the RLS+ and RLS- groups were performed using independent samples *t* test, Mann-Whitney *u* test and Pearson Chi square test where appropriate (*Table 1*). Afterwards, multivariate binary logistic regression models with enter and forward conditional methods were performed with the following steps:

1. Due to the large sample size, statistical power was so high that even small differences were detected as significant in univariate analysis. Therefore, among demographic variables, only the ones with a *p*-value of <0.05 in univariate comparison between RLS+ and RLS- groups that were also clinically relevant (based on expert judgment and literature) were selected as potential covariates. This list consisted of age, sex, level of education and smoking status. These four covariates were forced in all multivariate regression models.
2. For each comorbidity, a specific multivariate binary regression model was applied where the association between the comorbid condition and RLS was regressed out by the selected four covariates to calculate adjusted Odds’ ratio (OR) (*Table 2*).
3. In order to assess the independency of the association between each comorbidity and RLS, general multivariate binary regression models were run. In these models, the comorbidities of interest consisting of cardiometabolic burden, different neurocognitive variables and cancer were included as predictors. The four demographic covariates were also added to the models for further adjustment (*Tables 3 and 4*). Our strategy had three steps:
  - *Model 1*: an enter model including only the 4 control variables (age, sex, level of education and smoking status) as predictors of RLS
  - *Model 2*: an enter model including all 4 control variables and all comorbidities of interest as predictors of RLS
  - *Model 3*: a forward conditional method keeping all variables of model 2 into the regression model to predict RLS. For the stepwise criterion, the probability-to-enter and probability-to-remove were set as 0.05 and 0.1, respectively. The classification cutoff was considered as 0.5 with maximum 20 iterations. In the final step, the non-significant variables that were initially entered into the model were excluded.

In order to investigate the risk of collinearity between the predictors, we calculated and reported tolerance index, which represents the proportion of variance for each predictor that is not explained by other predictors in the model. The risk for collinearity was considered low if tolerance was >0.4 for each variable in the model.

**Table 1:** Socio-demographic and baseline characteristics of the study populations.

Variable	Total (n = 19176)	RLS- (n = 17596)	RLS+ (n = 1580)	*p*-value
**Age (yr)**- *Mean (SD)*	56.5 (10.2)	56.3 (10.1)	58.1 (10.2)	**<0.001**
**Gender**- *Number (%)*				
Female	12700 (66.2)	11604 (65.9)	1096 (69.4)	**0.006**
Male	6476 (33.8)	5992 (34.1)	484 (30.6)	
**Level of Education**- *Number (%)*				
Illiterate	3181 (17.0)	2829 (16.5)	352 (22.9)	
Primary school	5246 (28.0)	4767 (27.8)	479 (31.2)	
Secondary school	3317 (17.7)	3043 (17.7)	274 (17.8)	**<0.001**
High school/Diploma	4720 (25.2)	4412 (25.7)	308 (20.0)	
College/University	2243 (12.0)	2119 (12.3)	124 (8.1)	
**Marital Status**- *Number (%)*				
Single	310 (1.7)	294 (1.8)	16 (1.1)	
Married	14901 (81.8)	13693 (82.0)	1208 (79.8)	**0.001**
Widow	2655 (14.6)	2388 (14.3)	267 (17.6)	
Divorced	348 (1.9)	326 (2.0)	22 (1.5)	
**Occupation**- *Number (%)*				
Unemployed	566 (3.2)	520 (3.2)	46 (3.2)	
Employed	2782 (15.9)	2601 (16.2)	181 (12.4)	
Paid without working	318 (1.8)	284 (1.8)	34 (2.3)	**0.002**
Housekeeper	9432 (53.8)	8614 (53.6)	818 (56.1)	
Retired	3918 (22.4)	3591 (22.4)	327 (22.4)	
Others	510 (2.9)	457 (2.8)	53 (3.6)	
**Smoking**- *Number (%)*	1977 (10.3)	1775 (10.1)	202 (12.8)	**0.001**

RLS: restless legs syndrome; SD: standard deviation.

Comparisons have been performed by means of independent samples *t*-test or Pearson Chi square for numeric and nominal variables, respectively.

Statistical significant differences are highlighted with bold font (*p*-value<0.05).

**Table 2:** Co-morbidity profile of the study populations (n = 19176).

Co-morbidities *Number (%)*	Total (n = 19176)	RLS- (n = 17596)	RLS+ (n = 1580)	*Unadjusted OR (95% CI)*	*Adjusted OR (95% CI)*
Heart failure	2609 (13.6)	2279 (13.0)	330 (20.9)	**1.8 (1.6–2.0)**	**1.6 (1.4–1.8)**
Hypertension	5716 (29.8)	5101 (29.0)	615 (38.9)	**1.6 (1.4–1.7)**	**1.4 (1.2–1.5)**
Hyperlipidemia	5042 (26.3)	4446 (25.3)	596 (37.7)	**1.8 (1.6–2.0)**	**1.6 (1.5–1.8)**
Diabetes	3453 (18.0)	3106 (17.7)	347 (22.0)	**1.3 (1.2–1.5)**	**1.2 (1.1–1.4)**
Stroke	426 (2.2)	361 (2.1)	65 (4.1)	**2.0 (1.6–2.7)**	**1.7 (1.3–2.3)**
Headache	3431 (17.9)	3031 (17.2)	400 (25.3)	**1.6 (1.4–1.8)**	**1.6 (1.4–1.8)**
Back pain	5933 (30.9)	5239 (29.8)	694 (43.9)	**1.8 (1.7–2.1)**	**1.8 (1.6–2.0)**
Seizure	143 (0.7)	122 (0.7)	21 (1.3)	**1.9 (1.2–3.1)**	**1.7 (1.1–2.8)**
Parkinsonism	155 (0.8)	88 (0.5)	67 (4.2)	**8.8 (6.4–12.2)**	**7.4 (5.3–10.4)**
Acting out dreams	3108 (17.0)	2586 (15.4)	522 (35.0)	**3.0 (2.6–3.3)**	**2.8 (2.5–3.2)**
SCI	5404 (29.7)	4581 (27.4)	823 (54.7)	**3.2 (2.9–3.6)**	**3.1 (2.7–3.4)**
Peripheral neuropathy	5955 (32.3)	5000 (29.6)	955 (62.1)	**3.9 (3.5–4.4)**	**3.7 (3.3–4.1)**
Hyposmia	2228 (12.3)	1850 (11.1)	378 (25.4)	**2.7 (2.4–3.1)**	**2.5 (2.2–2.9)**
Cancers	196 (1.0)	169 (1.0)	27 (1.7)	**1.8 (1.2–2.7)**	**1.8 (1.2–2.7)**

RLS: restless legs syndrome; OR: odds’ ratio; CI: confidence interval; SCI: subjective cognitive impairment.

Univariate comparisons (unadjusted) have been performed by means of Pearson Chi square test.

Multivariate binary regression models with forward conditional method have been used to adjust comparisons for age, gender, level of education and smoking.

Statistical significant differences are highlighted with bold font (*p*-value<0.05).

**Table 3:** Multivariate binary logistic regression models to indicate independent determinants of restless legs syndrome in the entire study population.

Predictors	*Model 1 (Enter)*			*Model 2 (Enter)*			*Model 3 (Forward Conditional)*		
	OR (95% CI)	*Tolerance*	*p-value*	OR (95% CI)	*Tolerance*	*p-value*	OR (95% CI)	*Tolerance*	*p-value*
**Age**	**1.01 (1.01–1.02)**	0.806	**<0.001**	1.00 (0.99–1.01)	0.751	0.733	Excluded**	-	-
**Female Gender**	**1.40 (1.23–1.60)**	0.795	**<0.001**	1.12 (0.96–1.30)	0.772	0.146	Excluded	-	-
**Smoking**	**1.60 (1.34–1.91)**	0.838	**<0.001**	**1.52 (1.25–1.86)**	0.838	**<0.001**	**1.42 (1.19–1.70)**	0.992	**<0.001**
**Level of Education**									
Illiterate	1 (Reference)	-	-	1 (Reference)	-	-	1	-	-
Primary school	**0.86 (0.74–0.99)**	0.510	**0.042**	0.92 (0.78–1.09)	0.504	0.338	0.91 (0.77–1.07)	0.517	NS
Secondary school	**0.81 (0.68–0.96)**	0.550	**0.015**	0.92 (0.76–1.12)	0.543	0.412	0.91 (0.75–1.10)	0.580	NS
High school/Diploma	**0.64 (0.54–0.76)**	0.470	**<0.001**	**0.77 (0.63–0.93)**	0.461	**0.007**	**0.76 (0.63–0.91)**	0.511	**0.003**
College/University	**0.56 (0.45–0.69)**	0.598	**<0.001**	**0.74 (0.57–0.95)**	0.581	**0.018**	**0.72 (0.57–0.92)**	0.631	**0.009**
**Cardiometabolic Burden**									
0				1 (Reference)	-	**-**	1	-	**-**
1	Not Included*	**-**	-	**1.25 (1.07–1.45)**	0.828	**0.004**	**1.25 (1.08–1.45)**	0.843	**0.003**
2				**1.37 (1.15–1.63)**	0.816	**<0.001**	**1.38 (1.16–1.63)**	0.844	**<0.001**
3				**1.38 (1.11–1.71)**	0.868	**0.004**	**1.40 (1.11–1.72)**	0.890	**0.002**
4/5				**1.55 (1.13–2.13)**	0.927	**0.006**	**1.59 (1.16–2.25)**	0.944	**0.003**
**Neurocognitive Co-morbidities**									
Back pain				**1.29 (1.14–1.46)**	0.895	**<0.001**	**1.34 (1.19–1.52)**	0.945	**<0.001**
Parkinsonism				**2.73 (1.87–3.99)**	0.957	**<0.001**	**2.72 (1.87–3.97)**	0.960	**<0.001**
Headache				1.13 (0.98–1.30)	0.916	0.105	Excluded	-	-
Seizure	Not Included	**-**	-	1.21 (0.71–2.09)	0.991	0.485	Excluded	-	-
Acting out dreams				**1.55 (1.35–1.78)**	0.837	**<0.001**	**1.57 (1.36–1.80)**	0.841	**<0.001**
SCI				**1.83 (1.61–2.09)**	0.816	**<0.001**	**1.86 (1.63–2.11)**	0.822	**<0.001**
Peripheral neuropathy				**2.66 (2.35–3.01)**	0.866	**<0.001**	**2.67 (2.36–3.03)**	0.869	**<0.001**
Hyposmia				**1.40 (1.20–1.62)**	0.881	**<0.001**	**1.40 (1.20–1.63)**	0.883	**<0.001**
**Cancer**	Not Included	**-**	-	1.55 (0.97–2.47)	0.992	0.070	Excluded	-	-

B: regression coefficient; SE: standard error; OR: odds’ ratio; CI: confidence interval; NS: non-significant.

Statistical significant associations are highlighted with bold font (*p*-value<0.05). Any participant with a single missing value in any of the variables included in this multivariate regression model was excluded (missing rate = 15.9%, n = 16132).

*”Not included”: variables were not entered into the regression model from the beginning.

** “Excluded”: variables were indeed first included into the model but later excluded from the final step.

**Table 4:** Multivariate binary logistic regression models to indicate independent determinants of restless legs syndrome among those without peripheral neuropathy.

Predictors	*Model 1 (Enter)*			*Model 2 (Enter)*			*Model 3 (Forward Conditional)*		
	OR (95% CI)	*Tolerance*	*p-value*	OR (95% CI)	*Tolerance*	*p-value*	OR (95% CI)	*Tolerance*	*p-value*
**Age**	1.01 (0.99–1.02)	0.815	0.107	0.99 (0.98–1.01)	0.760	0.392	Excluded**	-	-
**Female Gender**	1.22 (0.99–1.50)	0.805	0.060	1.00 (0.80–1.25)	0.782	0.974	Excluded	-	-
**Smoking**	**1.58 (1.20–2.09)**	0.845	**0.001**	**1.41 (1.04–1.90)**	0.843	**0.027**	**1.37 (1.05–1.79)**	0.995	**0.021**
**Level of Education**									
Illiterate	1 (Reference)	-	-	1 (Reference)	-	-			
Primary school	1.05 (0.82–1.35)	0.482	0.701	1.16 (0.88–1.53)	0.479	0.298	Excluded	-	-
Secondary school	0.90 (0.68–1.21)	0.520	0.496	1.04 (0.76–1.43)	0.516	0.820			
High school/Diploma	**0.73 (0.55–0.97)**	0.432	**0.028**	0.85 (0.62–1.17)	0.427	0.316			
College/University	**0.54 (0.38–0.78)**	0.546	**0.001**	0.68 (0.46–1.02)	0.537	0.060			
**Cardiometabolic Burden**									
0				1 (Reference)	-	-	1	-	**-**
1	Not Included*	-	-	**1.50 (1.20–1.87)**	0.866	**<0.001**	**1.50 (1.20–1.86)**	0.902	**<0.001**
2				**1.78 (1.36–2.32)**	0.858	**<0.001**	**1.77 (1.37–2.29)**	0.913	**<0.001**
3				**1.48 (1.02–2.15)**	0.908	**0.040**	**1.48 (1.02–2.14)**	0.947	**0.037**
4/5				1.66 (0.92–3.00)	0.949	0.095	1.63 (0.91–2.92)	0.969	NS
**Neurocognitive Co-morbidities**									
Back pain				1.17 (0.96–1.44)	0.909	0.125	Excluded	-	-
Parkinsonism				**4.99 (2.13–11.7)**	0.981	**<0.001**	**4.64 (1.98–10.9)**	0.980	**<0.001**
Headache				**1.31 (1.04–1.64)**	0.932	**0.024**	**1.36 (1.09–1.71)**	0.980	**0.007**
Seizure	Not Included	-	-	0.41 (0.10–1.80)	0.989	0.238	Excluded	-	-
Acting out dreams				**1.66 (1.31–2.11)**	0.850	**<0.001**	**1.66 (1.31–2.11)**	0.849	**<0.001**
SCI				**1.86 (1.51–2.29)**	0.855	**<0.001**	**1.89 (1.53–2.32)**	0.868	**<0.001**
Peripheral neuropathy				Not Included	-	-	Not Included	-	-
Hyposmia				**1.74 (1.34–2.26)**	0.885	**<0.001**	**1.74 (1.34–2.25)**	0.885	**<0.001**
**Cancer**	Not Included	-	-	**2.20 (1.17–4.14)**	0.991	**0.015**	**2.21 (1.17–4.14)**	0.995	**0.014**

B: regression coefficient; SE: standard error; OR: odds’ ratio; CI: confidence interval; NS: non-significant.

Statistical significant associations are highlighted with bold font (*p*-value<0.05). Any participant with a single missing value in any of the variables included in this multivariate regression model was excluded (missing rate = 10.9%, n = 11119).

*”Not included”: variables were not entered into the regression model from the beginning.

** “Excluded”: variables were indeed first included into the model but later excluded from the final step.

We used IBM SPSS Statistics for Macintosh software, version 22.0 for all analytical procedures. A two-tailed *p*-value of <0.05 was considered as the threshold for statistical significant differences or associations.

## Results

### Baseline characteristics

Data were recruited from 19176 inhabitants consisted of 6476 (33.8%) males and 12700 (66.2%) females with the mean age of 56.5 (SD = 10.2) *yr* ranging between 30 and 95 *yr*. Detailed information on socio-demographic and baseline characteristics of the study population are summarized in *Table 1*.

### Prevalence of RLS

**Fig 1:**
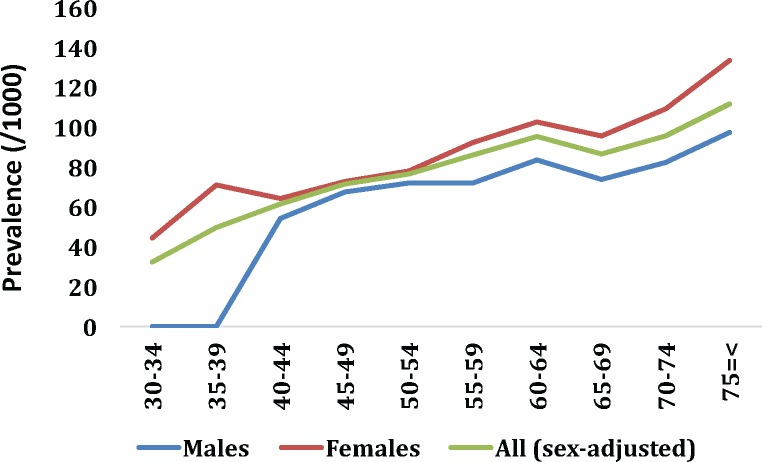
Prevalence rate (per 1000) of restless legs syndrome within different age-groups in each sex and total study population.

In total, 1580 individuals were positively screened for RLS who fulfilled the criteria by having all four symptoms. Crude prevalence rate was calculated as 8.2% (95% CI: 7.8%-8.6%). RLS was significantly more common in females [8.6% vs. 7.5%, OR = 1.2 (95% CI: 1.0–1.3), *p* = 0.006] with a gradual increase by advancing age. Prevalence of RLS increased by 3.5-fold from 3.2% in 30–34 *yr* age-group to 11.2% among those with ≥75 *yr* of age. As shown in *Fig 1*, a significant increasing trend in RLS prevalence was seen by advancing age in entire population and separately within each sex (all linear-by-linear trend *p*≤0.001). Age- and sex-adjustment by real Tehran population demography resulted in an adjusted prevalence rate of 60.0/1,000.

### Univariate determinants of RLS

We performed univariate comparisons of the baseline and socio-demographic characteristics between the individuals with (RLS+, n = 1580) and without RLS (RLS-, n = 17596) (*Table 1*). RLS+ group was significantly older [58.1 (SD = 10.2) *yr* vs. 56.3 (SD = 10.1) *yr*, *p*<0.001] and consisted of more females (69.4% vs. 65.9%, *p* = 0.006). Level of education was generally lower in the RLS+ group (*p*<0.001) and a higher proportion of them were active smokers compared to the RLS- group (12.8% vs. 10.1%, *p* = 0.001). *Table 2* shows the findings for univariate comparison of co-morbidity profile between the two groups. All comorbidities were significantly more prevalent in RLS+ group (all p<0.05) and the largest differences were observed in parkinsonism [unadjusted OR = 8.8 (95% CI: 6.4–12.2)], peripheral neuropathy [unadjusted OR = 3.9 (95% CI: 3.5–4.4)], SCI [unadjusted OR = 3.2 (95% CI: 2.9–3.6)] and acting out dreams [unadjusted OR = 3.0 (95% CI: 2.6–3.3)]. Cardiometabolic diseases were more prevalent among the individuals with RLS+ (Mann-Whitney *U*-test *p*<0.001).

### Multivariate determinants of RLS

As summarized in *Table 2*, all chronic conditions were more common in the RLS+ group even after multivariate adjustment. The largest difference was estimated for parkinsonism [adjusted OR = 7.4 (95% CI: 5.3–10.4)], peripheral neuropathy [adjusted OR = 3.7 (95% CI: 3.3–4.1)], SCI [adjusted OR = 3.1 (95% CI: 2.7–3.4)], acting out dreams [adjusted OR = 2.8 (95% CI: 2.5–3.2)] and hyposmia [adjusted OR = 2.5 (95% CI: 2.2–2.9)]. Multivariate binary logistic regression models were applied to indicate independent determinants of RLS in three steps. As it is shown in *Table 3*, results from both enter and forward conditional models to either force the all covariates and comorbidities or to keep only the significant ones in model are consistent with low risk of collinearity. Active smoking was accompanied with a higher odd of RLS [adjusted OR = 1.4 (95% CI: 1.2–1.7)], while higher education was found to be a protective factor [adjusted OR = 0.7 (95% CI: 0.6–0.9)]. Additional number of cardiometabolic diseases significantly increased the odd of having RLS with the highest risk among those with at least four cardiometabolic multimorbidities [adjusted OR = 1.6 (95% CI: 1.2–2.3)]. Following adjustment for other variables, parkinsonism [adjusted OR = 2.7 (95% CI: 1.9–4.0)], peripheral neuropathy [adjusted OR = 2.7 (95% CI: 2.4–3.0)], SCI [adjusted OR = 1.9 (95% CI: 1.6–2.1)], acting out dreams [adjusted OR = 1.6 (95% CI: 1.4–1.8)], hyposmia [adjusted OR = 1.4 (95% CI: 1.2–1.6)] and back pain [adjusted OR = 1.3 (95% CI: 1.2–1.5)] all increased the odd of RLS. In order to investigate the potential confounding effects of the mimicking conditions on co-morbidity profile of RLS, we performed a sensitivity analysis in which we repeated the same multivariate regression models in those without peripheral neuropathy (one of the most well-known mimickers of RLS). As shown in *Table 4*, the list of significant determinants of RLS is in line with that of *Table 3* in the entire population. Even after excluding 5955 participants with peripheral neuropathy, parkinsonism [adjusted OR = 4.6 (95% CI: 2.0–10.9)], SCI [adjusted OR = 1.9 (95% CI: 1.5–2.3)], acting out dreams [adjusted OR = 1.7 (95% CI: 1.3–2.1)], hyposmia [adjusted OR = 1.7 (95% CI: 1.3–2.3)] and cardiometabolic burden accompanied with higher prevalence of RLS.

Furthermore, we also investigated the effect of menopause on the prevalence of RLS and it failed to be a significant determinant factor after multivariate adjustment for demographic and co-morbidity profile in females [adjusted OR = 1.1 (95% CI: 0.88–1.27].

## Discussion

### Prevalence of RLS

To our knowledge, our study is the first population-based door-to-door survey to estimate the prevalence of RLS in Iran, in which 19176 individuals were screened using the minimal criteria of the IRLSSG. After age- and sex-adjustment, the prevalence rate of RLS was found to be 6%. Comparing to other studies with the same screening instrument, a prevalence of 6% for RLS could be considered a medium rate. Higher estimates range from 5.5% to 11% reported in adult Caucasian populations while prevalence rates are lower for Asian populations ranges from 1.0% to 7.5% [23]. In the Eastern Mediterranean WHO region, just one study with a smaller sample size (n = 2682) was performed, which reported a prevalence rate of 8.4% in general population in Saudi Arabia [24]. Unfortunately, there is a dearth of information from other Middle Eastern countries.

Our findings demonstrated that prevalence of RLS increases constantly by age, with a female preponderance in all age groups. According to previous literature, prevalence of RLS rises with age in European and North American countries but not in the Asian populations [2]. We observed a 3.5 times increase in RLS prevalence from the 30–34 year age group to those with 75 years or older. This finding is consistent with a survey conducted in five European countries in which a 2.5 times higher prevalence rate was shown in subjects with ≥50 years of age in comparison to those <40 years [25]. Our results are also in line with another large study with approximately 100,000 participants showing an increased risk of RLS with advancing age [26]. Based on a review article, some other studies have similarly reported an increase in RLS prevalence up to 60–70 years of age, however, the prevalence decreased in older age groups in some investigations [2]. Intriguingly, age failed to remain as an independent determinant of RLS following multivariate adjustment in our analysis. One possible explanation is that previous studies might not adjust for an inclusive list of age-related comorbid confounders. Prevalence of most of the health conditions that associate with RLS, namely cardiometabolic disorders and other neurologic illnesses, increases with age suggesting their confounding role in the relationship between advancing age and RLS.

According to our findings, RLS was slightly more prevalent among women (8.6% vs. 7.5%). Nevertheless, some previous studies reported a larger sex preponderance as high as twice in females [2] or even no difference [27]. Interestingly, female sex failed to remain as an independent determinant of RLS in our multivariate analysis after adjusting for co-morbidities. This is relatively consistent with previous studies in Arabian [24] and Turkish population [10]. Yet, there are other sex-related aspects susceptible for influencing RLS incidence such as current and past pregnancies [28]. In our study, we had data on menopause and it didn’t show up to associate with RLS after multivariate adjustment.

### Associated comorbidities

RLS may connote underlying neurodegenerative pathology, thereby majority of previous studies showed a significant association between RLS and comorbid parkinsonism [29]. In addition, investigators showed higher prevalence of RLS in people with Parkinson’s disease (PD) compared to general population [29, 30]. To our knowledge, current study is the first to investigate the relationship between RLS and parkinsonism in a large sample of general population. Remarkably, amongst all co-morbidities, parkinsonism demonstrated the strongest association with RLS. In one prospective cohort with a large sample size, researchers showed an increased risk of developing PD in men with RLS compared to those without RLS and even concluded that severe RLS might be a prodromal symptom of PD [31]. We showed a strong association between hyposmia and RLS even after adjustment for other comorbidities and the effect of aging. By contrast, some previous researches have shown that olfaction is not impaired in RLS [32].

Similar to our investigation, RLS was shown to significantly associate with neuropathies and radiculopathies by several other studies. Recently, RLS was found to be more prevalent in familial amyloid polyneuropathy related to transthyretin as compared to controls proposing that peripheral pathway play a part in RLS pathogenesis [33]. Our results showed that back pain was 30% more prevalent in individuals with RLS. According to previous evidence, people with RLS are at a higher risk of developing persistent painful conditions since the central nervous system for pain processing might be amplified [34].

In our study, heart failure demonstrated significant association with RLS. Likewise, components of metabolic syndrome such as hypertension, hyperlipidemia and diabetes were more prevalent among participants who were positively screened for RLS. In a systematic review, 13 out of 14 cross-sectional studies demonstrated varying degrees of association between RLS and cardiovascular diseases (CVD) [5]. One cross-sectional study demonstrated the so-called “dose-response” effect where a stronger association was observed between coronary artery disease and CVD with more severe RLS symptoms [35]. In another prospective study, having RLS for >3 years was linked to higher risk of coronary heart diseases [36]. On the other hand, researchers found that obesity and high cholesterol, but not hypertension, are significant risk factors for developing RLS in future [37]. Despite numerous studies demonstrated persistent association between RLS and either cardio-metabolic diseases or their risk factors, the underlying mechanisms and direction of causal relationships are not yet understood. Recent studies suggest that dysregulation of hypothalamic-pituitary-adrenal axis may play a key role in the link between RLS and CVD accompanied by unhealthy lifestyle factors, sleep disruptions and mood disorders [5]. Similar to our investigation, one study reported a significant positive association between the number of cardiovascular risk factors (vascular comorbidity index) and incidence of RLS [38]. Even after adjustment for cardio-metabolic burden and other covariates, active smoking remained to be a significant associated factor for RLS in our study. Opposite to PD, active smokers were 50% more likely to suffer from RLS. In line with our results, smoking was shown as a risk factor for RLS in another study [27].

### Study limitations and strengths

Definition of RLS in our study was based on the four-symptoms of the minimal criteria recommended by the IRLSSG [22]. Having no objective polysomnography data and information on frequency and severity of symptoms might have resulted in relatively high false positive rates [39]. Nonetheless, using the minimal IRLSSG criteria is the most feasible approach for the estimation of the prevalence of RLS in large-sample community-based surveys. The specificity of the IRLSSG criteria has been estimated as 84% since there are some mimicking conditions that are not distinguishable by the four items in the criteria [39]. Peripheral neuropathy, fibromyalgia, cramps, positional discomfort and anxiety disorders are the commonest mimicking conditions, which might have resulted in overestimation of the prevalence rate of RLS in our study. Considering the availability of data, we were able to explore the potential confounding effect of one of these mimicking conditions, peripheral neuropathy. Findings from sensitivity analysis showed almost the same co-morbidity profile for RLS in the sub-population without peripheral neuropathy. However, other important mimics for RLS are leg cramps and positional discomfort, which have not been considered in our study and should be mentioned as potential confounders.

Since our study lacked a confirmatory investigation phase by neurologists, we could not differentiate idiopathic and secondary RLS. In addition, the nature of some of the questions we used for comorbid conditions are not as valid as their gold standard measurement method. For instance, asking about acting on dreams during sleep by a single question could not validly replace laboratory-based polysomnography information. Nevertheless, self-reported data on medical records including comorbidity profile has been demonstrated as a valid proxy in settings where medical claims and administrative data are not available. This is the case in many community-based large-scale studies in poor-resource settings without electronic registration system [40]. As another limitation, we should mention that there are some other factors that might have influenced the prevalence of RLS such as obesity [26, 37] and pregnancy [28], which were neglected in our study. However, one should consider that our research was designed as a community-based study with large sample-size where data needed to be gathered in a timely manner during door-to-door visits. Often, in this type of data collection, interviewers do not have enough time to do anthropometric measurements by a standard procedure. Also, the list of questions and items were selected strategically to cover the most important symptoms/diseases in the limited time frame of such interview at each door. As a result, we inevitably missed data on some other aspects such as medication for instance. Despite all these limitations, our community-based study is one of the few investigations on the prevalence of RLS with an admissible sampling method in a huge urban area with a large sample-size. The comprehensive list of comorbidities -some of which haven’t been evaluated adequately so far- has also strengthened our results. A systematic review demonstrated that current evidence on the associated co-morbidities of RLS and the methodology of many of the previous studies on this topic are poor and inconclusive [41]. Thus, we hope that our findings could add more evidence and further enlighten the field.

## Conclusion

To our Knowledge, this is the first population-based study in Iranian population to evaluate prevalence of RLS using the minimum IRLSSG diagnostic criteria. In conclusion, RLS affected nearly 6% of total population with >30 years of age. Neuro-cognitive co-morbidities such as parkinsonism, peripheral neuropathy, SCI, acting out dreams during sleep, hyposmia and headache, as well as cardio-metabolic risk factors and diseases, smoking and level of education were all shown to be independent determinants of RLS. Our results are aligned with those of a recent systematic review concluding that cardiovascular disease, hypertension, diabetes, migraine, and PD might associate with RLS according to current literature [41]. Although further studies are needed to explore the directionality of these associations, it is reasonable to screen individuals with either of these comorbid conditions for RLS or the ones with RLS for the accompanying diseases. The rather medium prevalence rate is noteworthy raising notion for national health system administrators to increase awareness about RLS among physicians and healthcare workers. They should be trained about diagnosis, appropriate interventions and life style modifications in individuals with RLS since they confront serious distresses such as sleep disturbance, devastating quality of life and even an increased risk of mortality [42].

## Supporting information

S1 Data Data sheet. Case-wise data points in study population.
(XLSX)
